# Assisted Reproductive Technologies (ART) and breast cancer risk: hormonal treatments under the spotlight

**DOI:** 10.1007/s10549-026-08036-x

**Published:** 2026-07-29

**Authors:** Cristiana Boldrini, Micol Bottalico, Francesca L. Lia, Valentina Longo, Silvia Gigli

**Affiliations:** 1 Department of Bioimaging, Radiation Oncology and Hematology, Fondazione Policlinico Universitario A. Gemelli IRCSS, Largo A. Gemelli 8, 00168 Rome, Italy; 2 Department of Diagnostic Imaging, Sandro Pertini Hospital, Rome, Italy

**Keywords:** Assisted reproductive technologies, Breast cancer risk, Gonadotropins, Progesterone, Clomiphene, Infertility treatment, Hormonal exposure

## Abstract

**Background:**

Assisted Reproductive Technologies (ART) have become nowadays a cornerstone in the management of infertility. While these treatments are generally considered safe, concerns have been raised regarding their potential influence on breast cancer (BC) risk, particularly in specific subgroups of women.

**Objective:**

This narrative review aims to evaluate the most relevant past and current literature on the association between ART-related hormonal treatments and breast cancer risk, highlighting potential biological mechanisms, epidemiological evidence, and clinical implications.

**Methods:**

A literature search was conducted through PubMed, focusing on medium-to-large cohort and case-control studies, meta-analyses, and guidelines published to date. Evidence was synthesized by distinguishing overall ART/IVF risk estimates from drug-specific findings and subgroup-specific risk modifiers. Particular attention was given to the most recent or most comprehensive reports when multiple publications were derived from the same cohort.

**Results:**

To date, current evidence does not support a real and consistent overall increase in breast cancer risk following ART-related hormonal treatments. Large population-based studies and recent meta-analyses are generally reassuring, particularly when ART-treated women are compared with infertile untreated women rather than with the general population. Some studies report modest risk elevations in selected subgroups, including nulliparous or nulligravid women, women undergoing repeated or high-dose stimulation cycles, and women with hereditary BC predisposition; however, these findings are inconsistent and often limited by residual confounding, small subgroup numbers, and incomplete dose information. Progesterone supplementation appears safe in the context of ART, with no consistent evidence of increased BC risk. Gonadotropins, including hMG, may transiently increase estrogen exposure, but large-scale studies and meta-analyses generally do not confirm a long-term increase in breast cancer incidence.

**Graphical Abstract:**

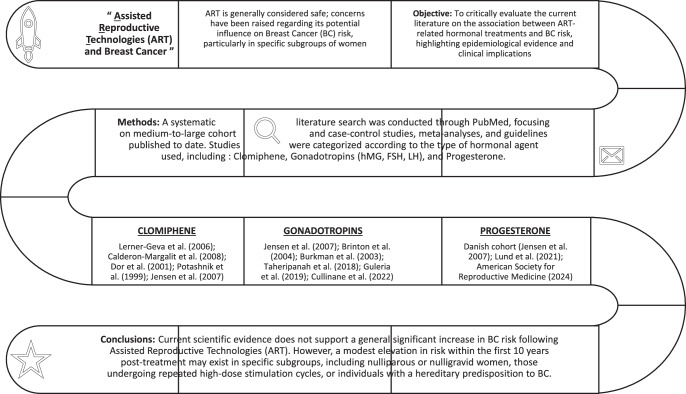

## Highlights

- Assisted reproductive technologies (ART) are generally considered safe
- Some concerns have been raised regarding their potential influence on breast cancer (BC) risk
- This narrative review evaluated the most relevant literature on the association ART - BC risk
- Studies were synthesized according to the type of evidence: overall ART/IVF risk, drug-specific exposure, and subgroup-specific modifiers
- Subgroup analysis also considered parity, cumulative exposure, family history and genetic predispositions
- Current evidence does not support a consistent general increase in BC risk following ART, although small subgroup-specific risks cannot be excluded

## Introduction

Over the past three decades, the use of assisted reproductive technologies (ART) has expanded dramatically worldwide, reflecting both social and medical advances that have enabled many couples to overcome infertility. Infertility is now recognized as a major global health issue: according to the World Health Organization (WHO), approximately one in every six adults worldwide (≈17.5%) experiences infertility at some point in their reproductive lifetime. More specifically, lifetime infertility prevalence has been estimated at 17.8% in high-income countries and 16.5% in low- and middle- income countries [1,2]. Similarly, the European Society of Human Reproduction and Embryology (ESHRE) reports that infertility lasting at least 12 months affects 8–12% of women aged 20–44 years globally [3]. Given this substantial prevalence, an increasing number of women are exposed to fertility treatments—and consequently, to potential long-term health implications related to hormonal exposure. This growing exposure has raised ongoing concerns about possible adverse long-term effects, particularly the risk of developing hormone-sensitive malignancies. Among these, breast cancer has been the most frequently investigated, followed by ovarian and endometrial cancers. These tumors are of special interest because their development is closely linked to hormonal regulation, which may be significantly altered during fertility treatment.

Importantly, infertility itself has been associated with hormonal and metabolic alterations, such as anovulation, prolonged exposure to unopposed estrogens, and endocrine disorders like polycystic ovary syndrome (PCOS). These intrinsic risk factors complicate the interpretation of studies evaluating cancer risk after ART, as it remains difficult to disentangle the oncologic impact of infertility per se from that of pharmacologic ovarian stimulation. Moreover, women undergoing ART often undergo closer medical surveillance than the general population, which may introduce detection bias and lead to an apparent increase in cancer diagnoses.

A further methodological issue concerns the choice of the reference population. Women who conceive after successful ART may have a transiently increased risk of breast cancer related to recent childbirth rather than to ART exposure itself. Nichols et al. in 2019 showed that, compared with nulliparous women, parous women younger than 55 years had a breast cancer risk that peaked approximately five years after childbirth and then gradually declined over time [4]. Previous studies had already reported that recent childbirth confers a short-term increase in breast cancer risk, which may last 10 or more years and be amplified in women who are older at first birth [5]. The authors used data from the Premenopausal Breast Cancer Collaborative Group, a pooled project involving 20 prospective cohort studies [6]. When breast cancer incidence in ART-treated women is compared with that of the general female population, which includes both parous and nulliparous women, the risk may appear artificially increased if recent childbirth is not adequately accounted for. This bias may be small in some cohorts, but it could partly explain modest short-term increases in breast cancer incidence observed after ART. For this reason, studies using infertile untreated women or appropriately adjusted parous controls may provide more reliable estimates than comparisons with the general population alone.

From a biological perspective, the link between ovarian stimulation and carcinogenesis is theoretically plausible. Fertility drugs such as clomiphene citrate, gonadotropins, and progesterone act primarily through hormonal mechanisms that modify the hypothalamic–pituitary–ovarian axis, resulting in substantial fluctuations in circulating estrogen and progesterone levels. Estrogen exposure has long been implicated in breast carcinogenesis through multiple mechanisms: it promotes cellular proliferation, induces DNA damage via reactive metabolites, and modulates gene expression through estrogen receptor activation [7]. Similarly, progesterone has been shown to influence breast epithelial proliferation and differentiation, although its precise role in tumorigenesis remains debated. The combination of repeated ovarian stimulation and exogenous hormone administration has therefore prompted concerns regarding a potential cumulative effect on breast tissue over time [8,9].

Epidemiological studies have produced conflicting results. Early investigations suggested a possible increased risk of breast cancer following the use of ovulation-inducing agents, particularly after repeated treatment cycles or high cumulative drug doses. However, more recent large-scale cohort studies and meta-analyses have found no consistent evidence of an increased risk of breast cancer in women exposed to ART compared with infertile women who did not undergo such treatment. Nevertheless, the variability in study design, population characteristics, drug protocols, and duration of follow-up continues to limit the ability to draw definitive conclusions [10].

In 2008, it was estimated that approximately 10% of couples in developed countries used medically assisted reproductive techniques (ART) [11,12]. By 2004, about 1% of children were born through ART, and between 2004 and 2010, roughly 7.4 million American women underwent infertility treatments [11]. The duration of exposure to estrogen and progesterone plays a key role in breast cancer (BC) risk, as evidenced by associations with late first pregnancy, early menarche, late menopause, and long-term hormone therapy use [13]. Consequently, whether fertility drugs influence breast cancer incidence has become a topic of considerable interest. Given the increasing use of ART, the rising average maternal age, and the overlap of infertility with established breast cancer risk factors, clarifying the relationship between fertility treatments and oncologic risk is a matter of both scientific and clinical relevance. Understanding this association is essential for evidence-based patient counseling, risk–benefit evaluation, and the long-term safety assessment of ART protocols.

This review aims to provide a comprehensive synthesis of the current evidence on the potential link between assisted reproductive technologies (ART) and breast cancer risk, with particular emphasis on the use of clomiphene, gonadotropins, and progesterone. We critically examined epidemiological, clinical, and mechanistic studies, identifying subgroups potentially at increased risk, evaluating methodological limitations, and highlighting gaps in literature. The goal is to offer a clear, evidence-based perspective to guide clinical decision-making and inform future research in reproductive medicine.

## Methods

A comprehensive literature search was conducted in the PubMed® database to identify studies evaluating the association between assisted reproductive technologies (ART) and the risk of female cancers, with particular focus on breast cancer. The search was performed up to May 2025 and included a combination of Medical Subject Headings (MeSH) and free-text terms such as “assisted reproductive technologies,” “ART,” “in vitro fertilization,” “IVF,” “ovarian stimulation,” “clomiphene,” “gonadotropins,” “FSH,” “LH,” “hCG,” “progesterone,” and “breast cancer risk”. No language restrictions were applied at the initial screening stage, but only studies published in English were included for full-text review. Reference lists of selected papers and recent systematic reviews were also manually screened to identify additional relevant studies not captured by the initial database search.

Titles and abstracts of all retrieved records were independently screened by two reviewers to assess eligibility, and full texts were obtained for all potentially relevant articles. Studies were included if they were original cohort, case–control, or nested case–control studies evaluating the association between ART or specific fertility drugs (clomiphene, gonadotropins, or progesterone) and the risk of breast or other female cancers. Eligible studies were required to report effect estimates, including odds ratios, relative risks, or hazard ratios, with corresponding 95% confidence intervals, and to include a comparison group of infertile women not exposed to fertility drugs or the general female population. Studies with fewer than 1000 participants, follow-up shorter than five years, case reports, case series, editorials, commentaries, conference abstracts, or non-human studies were excluded. When multiple publications used the same cohort, the most recent or comprehensive study was selected for inclusion.

Data from eligible studies were independently extracted by two authors using a standardized data collection form to ensure consistency and accuracy. For each study, bibliographic details such as author, year, and country of publication were recorded, along with study design and sample size. Detailed information on fertility drug exposure was collected, specifying the type of drug, cumulative dose, and the number of treatment cycles. Population characteristics, including age at treatment, underlying cause of infertility, and parity, were also extracted, together with follow-up duration and timing of outcome assessment. Reported outcomes, including incidence of breast cancer and effect estimates with corresponding confidence intervals, were carefully documented. Given the heterogeneity in study design, exposure definitions, and outcome reporting, a qualitative synthesis was performed rather than a formal meta-analysis. Studies were grouped according to the type of fertility drug evaluated and whether outcomes pertained specifically to breast cancer or to female cancers more broadly. It should be noted that this work is a **narrative review**, and not a systematic review registered in PROSPERO.

The methodological quality and risk of bias of the included studies were assessed using the Newcastle–Ottawa Scale (NOS) for observational studies, which evaluates selection of study groups, comparability of exposed and unexposed groups, and ascertainment of outcomes. Each study was scored on a 0–9 scale, with scores of seven or higher considered indicative of high methodological quality. Discrepancies in quality assessment between reviewers were resolved by consensus, with arbitration by a third author when necessary. Common limitations identified across studies included short follow-up periods, inadequate adjustment for those potential confounders such as body mass index and family history, and heterogeneity in the definitions of drug exposure.

**Fig. 1:**
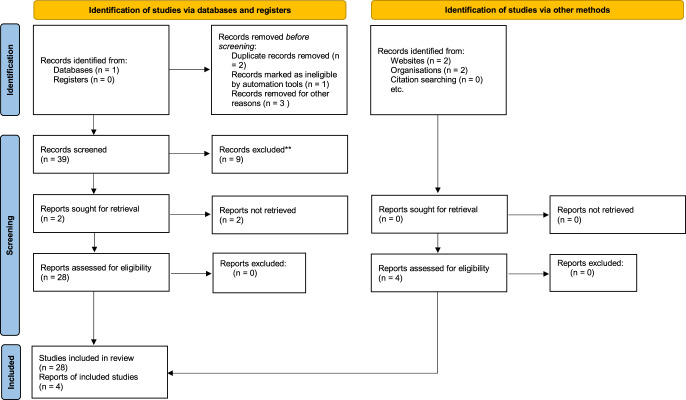
PRISMA 2020 flow diagram that maps out the number of records identified, included and excluded, and the reasons for exclusions for this review. This work is licensed under CC BY 4.0 from Page MJ, et al. BMJ 2021;372:n71. 10.1136/bmj.n71 [14]. To view a copy of this license, visit https://creativecommons.Org/licenses/by/4.0/

As this review was based on previously published data, no ethical approval or informed consent was required. Although this work is a narrative review and not a PROSPERO-registered systematic review, a PRISMA 2020 flow diagram was used, where applicable, to transparently report the study selection process (Fig. 1) [14].

## Results

The PubMed® search identified a total of 28 medium-to-large studies investigating the relationship between fertility treatments and breast cancer risk. Among these, 9 studies specifically addressed clomiphene citrate (CC), 6 focused on gonadotropins, and 4 on progesterone. The remaining 9 studies examined breast cancer risk in the context of assisted reproductive technologies (ART) more generally, considering all categories of ovulation-inducing drugs.

To address the heterogeneity of the available literature, the Results section has been reorganized to separate overall ART/IVF risk estimates from drug-specific evidence and subgroup-specific risk modifiers. This structure avoids assigning mixed-exposure studies to a single drug category when the original study design did not allow such attribution.

### Overall breast cancer risk after ART or fertility treatment

Large-scale studies evaluating ART or fertility treatment as a whole provide the most appropriate framework for interpreting breast cancer risk, because many cohorts include mixed drug exposures and do not allow reliable attribution to a single hormonal agent. Venn et al. [15] reported no overall excess of breast or ovarian cancer after IVF-related fertility drug exposure, although a transient increase in breast and uterine cancer diagnoses was observed within the first year after treatment, possibly reflecting short-term surveillance or detection effects rather than a causal long-term association.

The large British population-based linkage study by Williams et al. [16], including more than 266,000 women treated with assisted reproduction, found no overall increase in breast cancer risk. A small increase in in situ breast cancer was reported and was associated with the number of treatment cycles, but no significant excess was observed for invasive breast cancer. These findings suggest that intensified medical surveillance and screening after ART may partly contribute to the detection of early or in situ lesions.

More recent studies and reviews have generally supported a reassuring interpretation. Diakosavvas et al. [17] emphasized that infertility and cancer share several potential confounders, including anovulation, nulliparity, obesity and smoking. Vassard et al. [18] reported a modest increase in breast cancer risk after ART, particularly among women initiating treatment at age 40 years or older; however, interpretation requires caution because age at first birth, parity, recent childbirth and underlying infertility may all influence breast cancer risk independently of ART exposure.

Recent reviews and meta-analyses further support the absence of a consistent overall association. Furlong et al. [19] concluded that most studies do not show a significant increase in breast cancer risk after either injectable or oral fertility medications. Liu et al. [20] found no significant increase among women with a family history of breast cancer or BRCA mutations. Mihai et al. [21] similarly concluded that IVF does not appear to significantly increase overall breast cancer risk, while individual factors such as age, genetic predisposition, hormone exposure level and breast density may modify risk. Finally, the 2024 meta-analysis by Chen et al. [22] found no statistically significant difference in breast cancer risk between exposed and unexposed women, although ovarian cancer risk was increased after ovarian stimulation, supporting the need for site-specific interpretation of female cancer outcomes.

Overall, studies evaluating ART as a broad exposure are generally reassuring for breast cancer, but modest increases reported in selected settings should be interpreted in light of residual confounding, comparison-group selection, time since childbirth and detection bias.

### Drug-specific evidence: clomiphene citrate (CC) and breast cancer risk

Among ovulation-inducing drugs, clomiphene citrate (CC) and human menopausal gonadotropins (hMG) have been used since the 1960s, although they are now partly replaced by recombinant follicle-stimulating hormone [13]. CC remains the first-line treatment for women with anovulatory cycles [23]. Its mechanism of action is based on its structural similarity to estrogen, allowing it to bind to estrogen receptors (ERs) in the hypothalamic-pituitary axis, producing receptor saturation [23,24]. This reduces receptor availability and, due to CC’s weak estrogenic effect, inhibits the normal negative feedback mechanism. As a result, gonadotropin-releasing hormone (GnRH) secretion increases, elevating the frequency of follicle-stimulating hormone (FSH) and luteinizing hormone (LH) surges in normally menstruating women [23]. CC reduces hypothalamic ER concentration, diminishing negative feedback and stimulating gonadotropin production, which ultimately raises circulating gonadotropin levels.

Despite decades of use in anovulatory women, the link between CC and breast cancer remains debated. The evidence should be interpreted cautiously because several studies included mixed exposures, small exposed subgroups, and different comparator populations. Accordingly, the most recent and most comprehensive reports are prioritized when the same cohort produced multiple publications. The link between CC and breast cancer has been debated, with studies showing conflicting results. Some research suggests increased BC risk following CC treatment [24], others indicate a reduced risk [25], or no effect at all [26]. Some studies suggest CC may be the only fertility drug associated with higher BC risk [27], while factors such as the interval since stimulation [28] or intensive medical monitoring of study populations [29] may also influence outcomes. To clarify, the literature from the last 20 years can be grouped into three categories: studies suggesting a predisposition to BC, studies suggesting protection, and studies indicating no correlation.

Lerner-Geva et al. (2006) reported an increased risk among CC-treated women compared with untreated women (HR = 1.49; 95% CI: 1.15–1.93) [24]. However, this association should be interpreted with caution because residual confounding by infertility characteristics, parity, treatment indication, and medical surveillance cannot be excluded. The finding is therefore best considered as a signal from an observational cohort rather than evidence of a direct causal effect of CC. Importantly, this increased risk persisted even when controlling traditional BC risk factors, suggesting a potential direct influence of CC. While higher gonadotropin levels from CC were not directly linked to BC risk, evidence from animal studies suggests that LH-induced ovarian hyperstimulation could lead to mammary hyperplasia and cancer predisposition [30]. CC may affect BC risk by both blocking ERs (anti-estrogen effect) and inducing transcriptional activity (estrogenic effect) [31].

Potashnik et al. (1999) performed a long-term follow-up of 1,197 infertile women over 17.9 years [25]. Among 780 women treated with CC and/or hMG, 38 cancers were identified, including 20 breast cancers (SIR 1.40; 95% CI: 0.83–2.10), indicating a non-significant increase compared to the general population. Interestingly, the highest SIR for BC (2.6; 95% CI: 1.19–5.0) occurred in patients with only one or two CC cycles, mostly detected within five treatment cycles and cumulative doses ≤ 2,000 mg, suggesting fertility drugs were unlikely contributors to cancer development.

Jensen et al. (2007) evaluated 54,362 infertile Danish women for BC risk related to fertility drugs [26]. Median follow-up was 8.8 years. CC was the most used drug (31% cases; 33% subcohort), followed by hCG, gonadotropins, GnRH, and progesterone. During follow-up, 331 women developed invasive BC (median age 44). After adjusting for reproductive factors, no significant associations were found between BC risk and CC, gonadotropins, hCG, or GnRH. Only progesterone use was associated with increased BC risk (RR 3.36; 95% CI: 1.60–7.07).

Calderon-Margalit et al. (2008) evaluated ovulation-inducing treatments in a population-based cohort of 15,426 parous women in Jerusalem [27]. Increased risks were reported after ovulation induction treatment overall and after CC exposure, particularly among women who conceived after more than 12 months. However, because infertility duration, treatment indication and parity-related factors may influence breast cancer risk, these findings should be interpreted as subgroup-specific and hypothesis-generating.

Dor et al. (2001) studied cancer incidence in 5,026 women undergoing IVF [28]. The mean age at first IVF was 34 ± 6.4 years, with follow-up ending at 37.5 ± 7.1 years. Twenty-seven cancers were observed versus 35.6 expected (Standardized Incidence Ratio [SIR] 0.76; 95% CI: 0.50–1.10), with breast being the most common site. Although women with anovulatory infertility showed a 40% increase in BC risk, no significant differences emerged between treated and untreated women, suggesting that cancer may be influenced by hormonal changes rather than ovulation induction itself (Table 1).

**Table 1:** Clomiphene and breast cancer (BC) risk. The table provides an overview of the works cited in the present review article on the association between breast cancer risk and the use of **Clomiphene** in medically assisted procreation, with summary details on the number of patients involved, the duration of follow-up and finally some notes on the main findings

Study	Year	Population	Exposure	Follow-up	Main Findings	Notes
Potashnik et al. [25]	1999	1,197 infertile women	Clomiphene (and/or hMG)	17.9 ± 5 years	Non-significant increase of BC risk compared to the general population (SIR 1.40; 95% CI: 0.83–2.10)	The highest SIR for BC (2.6; 95% CI: 1.19–5.0) occurred in patients with only one or two CC cycles
Dor et al. [28]	2001	5,026 women undergoing IVF	Clomiphene	15 years	No significant differences emerged between treated and untreated women (Standardized Incidence Ratio [SIR] 0.76; 95% CI: 0.50–1.10)	Women with anovulatory infertility showed a 40% increased risk
Burkman et al. [32]	2003	Case-control	Clomiphene	-	No overall increased risk; some risk for <6 months or for fewer than six treatment cycles	Ductal BC slightly elevated; in the overall cohort, infertility drug use was not associated with a higher incidence of invasive ductal or lobular carcinoma
Brinton et al. [29,33]	2004	12,193 infertile women	Clomiphene	20+ years	Slight increase in BC risk after long-term follow-up	Risk higher in nulliparous women
Lerner-Geva et al. [24]	2006	3,076 women with known treatment cycles	Clomiphene (and/or hMG)	20.9 years	Multivariate analysis revealed that CC-treated women had an elevated risk compared to untreated women (Hazard Ratio [HR] = 1.49; 95% CI: 1.15–1.93)	Women with early menarche, 3–5 years of infertility, non-hormonal infertility, and CC-treated women had higher risk
Jensen et al. [26]	2007	54,362 infertile women	Clomiphene	8.8 years	After adjusting for reproductive factors, no significant associations were found between BC risk and CC (also: gonadotropins, hCG, or GnRH); slight risk increase in nulliparous women after multiple cycles	Large cohort study
Calderon-Margalit et al. [27]	2008	15,426 parous women	Clomiphene (and hMG)	30 years	Women receiving any ovulation induction treatment had a significantly increased BC risk. Women treated only with CC had an HR of 1.74 (95% CI: 1.09–2.79), and those who conceived after more than 12 months had an HR of 2.82 (95% CI: 1.40–5.65)	Those patients who conceived after more than 12 months had even higher risk
Meta-analysis (Beebeejaun et al.) [34]	2021	-	Clomiphene	-	No general significant increase in BC risk	Included various infertile populations

### Drug-specific evidence: gonadotropin-based ovarian stimulation, including hMG, FSH and LH

Gonadotropins, including human menopausal gonadotropin (hMG), follicle-stimulating hormone (FSH) and luteinizing hormone (LH), are central to controlled ovarian stimulation protocols in ART. Because hMG is itself a gonadotropin-containing preparation, the evidence on hMG is discussed within the broader gonadotropin section rather than as a separate category. This organization better reflects the structure of the published literature, in which treatment protocols often include mixed gonadotropin exposure.

Biologically, gonadotropins do not act directly on breast tissue, but they can markedly increase circulating estrogen levels during stimulated cycles. This creates a plausible theoretical concern for proliferative effects in hormone-sensitive breast tissue. Experimental data also suggest that high-estrogen environments during ovarian stimulation may influence gene expression, DNA repair pathways, imprinting and methylation patterns [35]. Nevertheless, biological plausibility alone is insufficient to establish clinical risk, and epidemiological evidence remains the key basis for interpretation.

In the large Danish cohort by Jensen et al. (2007), no overall increase in breast cancer incidence was observed after gonadotropin exposure, although a modest elevation was reported among nulliparous women [26]. Earlier findings from the U.S. infertility cohort suggested possible long-term associations after ovulation-stimulating drugs; however, the updated 30-year analysis by Brinton et al. (2014) provides the most complete evidence from this cohort and is therefore considered the primary source for interpretation. In that updated report, gonadotropin use was associated with invasive breast cancer among nulligravid women (HR 1.98; 95% CI, 1.04–3.60), whereas no overall association was observed in the full cohort [33]. This pattern suggests that underlying infertility, absence of pregnancy and subgroup characteristics may be more relevant than gonadotropin exposure alone.

Case-control studies have reported possible risk elevations after prolonged hMG exposure. Burkman et al. found moderately increased risks among women treated with hMG for more than six months or at least six cycles [32], and Taheripanah et al. reported an increased risk among women exposed to hMG for longer durations, while shorter exposure was not associated with breast cancer risk [11]. These findings are limited by incomplete cumulative dose information and by the difficulty of separating treatment effect from severity and duration of infertility.

In contrast, large registry-based investigations and meta-analyses are generally reassuring. Guleria et al. (2019), with more than 20 years of median follow-up, found no significant overall association between gonadotropin exposure and breast cancer [36]. Sergentanis et al. (2014), Beebeejaun et al. (2021), and Cullinane et al. (2022) similarly did not identify a significant increase in breast cancer risk after controlled ovarian stimulation or gonadotropin exposure [34,37,38]. Subgroup findings in women with first-degree family history or BRCA1/2 mutations remain limited and statistically uncertain [39,40]. Limitations across these studies include heterogeneity, variable follow-up duration, incomplete adjustment for confounding factors, and insufficient detail regarding specific drugs and dosages [41].

Interestingly, some data suggest a potential protective effect. Orgéas et al. (2009) reported a reduced BC risk among exclusive gonadotropin users, albeit with limited statistical power [42]. Experimental studies in rodents by Russo et al. have demonstrated that administration of human chorionic gonadotropin (hCG) to virgin rats led to a dose-dependent reduction in mammary tumor incidence and multiplicity, suggesting a possible hormonal mechanism for BC prevention [43].

Overall, current evidence does not support a consistent increase in breast cancer risk after gonadotropin-based ovarian stimulation, including hMG. Reported increases are largely confined to selected subgroups, such as nulliparous or nulligravid women or those with prolonged exposure, and should be interpreted in light of residual confounding, incomplete dose-data and comparator-group limitations (Table 2).

**Table 2:** Gonadotropin-based ovarian stimulation, including hMG, FSH and LH, and BC risk. Summary of studies evaluating **Gonadotropin**-based ovarian stimulation, including hMG when reported, and breast cancer risk. Because hMG is a gonadotropin-containing preparation, it is integrated in this table rather than presented as a separate drug category

Study	Year	Population	Exposure	Follow-up	Main Findings	Notes
Jensen et al. [26]	2007	54,362 infertile women	Gonadotropins	8.8 years	No overall increase; slight risk in nulliparous women	Cohort study
Brinton et al. [29,33]	2004	12,193 infertile women	Gonadotropins	20+ years	Invasive BC risk increased among nulligravid women	Long-term follow-up; the risk was most pronounced in nulliparous women and in those receiving high cumulative doses of gonadotropins (≥65 ampules); no overall association was observed in the full cohort
Burkman et al. [32]	2003	Case-control	Gonadotropins	-	Risk elevated with hMG > 6 months or ≥ 6 cycles	Ductal BC slightly elevated; in the overall cohort, infertility drug use was not associated with a higher incidence of invasive ductal or lobular carcinoma
Taheripanah et al. [11]	2018	Case-control	Ovulation induction drugs	<6 months	No overall increased risk; hMG > 6 months showed moderate increase	Limited cumulative dose data
Guleria et al. [36]	2019	1.3 million women	Gonadotropins	Median 20.9 years	No significant association overall. Compared with fertile women, infertile women who had used any fertility drugs did not have an increased hazard for breast cancer (BC) overall (HR = 1.02; 95% CI, 0.95–1.10), and none of the specific types of fertility drugs affected the overall hazard for breast cancer.	Population-based cohort (nationwide cohort of Danish women). No associations between use of any and specific types of fertility drugs and histological types (ductal, lobular, and mucinous) of BC.
Cullinane et al. [38]	2022	Meta-analysis	Gonadotropins	-	No significant association with BC	Overall, 25 studies, including 617,479 participants, were eligible for inclusion. There was no significant breast cancer risk association with fertility treatment (compared with general and subfertility reference groups). Summary odds ratio of all included studies was 0.97 (95% c.i. 0.90–1.04).
French E3N cohort [39]	2004	92,555 (6,602 were treated for infertility)	Infertility treatment	10 years	The study showed no overall significant association between breast cancer (BC) risk and treatment for infertility (RR = 0.95, confidence interval 0.82–1.11), after surgery or fertility drugs (FDs), and whatever the type, the duration of use and the age at first use of FDs. Infertility treatment was associated with an increased risk, of borderline significance, of breast cancer among women with a family history of breast cancer.	No specific drug distinction
Kotsopoulos, Librach et al. [40]	2008	4,994 BRCA1/2 carriers	Gonadotropin-containing drugs	6.5 years	Possible increased risk, but not statistically significant; odds ratio [OR] = 1.21; 95% confidence interval [CI] = 0.81–1.82 compared to non-users.	High-risk population

### Drug-specific evidence: progesterone and luteal phase support

Assisted reproductive technologies (ART), particularly in vitro fertilization (IVF), have revolutionized infertility management. A central component of ART success is the preparation and maintenance of an optimal endometrial environment for embryo implantation [44]. Progesterone, a steroid hormone produced primarily by the corpus luteum and subsequently by the placenta, is crucial for establishing endometrial receptivity [45]. During the luteal phase of a natural menstrual cycle, rising progesterone levels transform the proliferative endometrium into a secretory one, supporting embryo implantation. Progesterone also suppresses further follicular development and modulates immune responses to promote embryo tolerance [46,47]. In ART cycles, however, luteal phase function is often disrupted due to ovarian stimulation or GnRH analog administration, leading to luteal phase deficiency (LPD). LPD results in suboptimal endometrial development and reduced implantation rates if not corrected [48]. Progesterone supplementation, typically initiated on the day of oocyte retrieval or embryo transfer and continued until placental progesterone production is adequate (around 10–12 weeks gestation), effectively counteracts this deficiency. Administration routes include vaginal, oral, rectal, subcutaneous, or intramuscular, with both natural and synthetic progestogens employed [49,50]. Evidence consistently shows that progesterone supplementation improves implantation and live birth rates, though optimal dose, route, and duration remain under investigation [51–53].

Progesterone also plays a pivotal role in breast tissue development and function. Acting through progesterone receptors (PR) expressed in mammary epithelial cells, it stimulates lobulo-alveolar development during puberty and pregnancy, often in concert with estrogen [54]. However, the relationship between progesterone and breast cancer is complex and context-dependent [55]. Progesterone exhibits mitogenic effects on breast epithelial cells and can, under certain conditions, contribute to tumorigenesis [56]. While endogenous progesterone during pregnancy is generally protective against breast cancer, exogenous progesterone, especially in hormone replacement therapy, has been associated with increased risk [57]. In hormone receptor-positive breast cancers, progesterone can stimulate cell proliferation via paracrine pathways, including RANKL and Wnt signaling [55].

Data on progesterone use in ART and breast cancer risk are generally reassuring, although early findings were mixed. Jensen et al. (2007) reported an increased risk among progesterone users, but only 13 breast cancer cases had been exposed to progesterone, most participants had also received other fertility drugs, and no dose-response relationship was identified [26]. This result should therefore be interpreted cautiously and should not be considered definitive evidence of a progesterone-specific effect.

Recent meta-analyses reinforce the lack of a significant association [38,58]. A 2022 meta-analysis of 25 studies published by Cullinane et al. [38] aimed to determine whether there is a causal relationship between different fertility treatments (clomiphene, human chorionic gonadotropin, gonadotropin analogues and progesterone) and breast cancer. Examination of specifical progesterone exposure reported a pooled odds ratio of 1.11 (95% CI: 0.76–1.61), indicating no statistically significant increase in breast cancer incidence, though substantial heterogeneity existed across studies (I^2^ = 86%, *p* < 0.001). The 2024 American Society for Reproductive Medicine (ASRM) guideline “Fertility Drugs and Cancer” also concluded that ART—including progesterone administration—does not appear to increase breast cancer risk [59].

Despite this generally reassuring evidence, certain subgroups require careful consideration. Breast cancer survivors, particularly those with hormone receptor-positive disease, may face elevated risks if exposed to hormone-based fertility treatments. Non-hormonal ART protocols, such as natural cycle IVF, are often preferred to minimize potential stimulation of hormone-sensitive tissues [60–63]. Women with BRCA1 or BRCA2 mutations also warrant particular attention, as altered progesterone metabolism or receptor function may theoretically heighten breast tissue susceptibility, although specific evidence remains limited [64–66]. Individualized fertility planning and oncologic consultation are strongly recommended in these high-risk populations (Table 3).

**Table 3:** Progesterone and BC risk. The table provides an overview of the works cited in the present review article on the association between breast cancer risk and the use of **Progesterone** in medically assisted procreation, with summary details on the number of patients involved, the duration of follow-up and finally some notes on the main findings

Study	Year	Population	Exposure	Follow-up	Main Findings	Notes
Danish cohort (Jensen et al.) [26]	2007	54,362 infertile women	Progesterone	8.8 years	After adjusting for reproductive factors, no significant associations were found between BC risk and CC, gonadotropins, hCG, or GnRH. Only progesterone use was associated with increased BC risk (RR 3.36; 95% CI: 1.60–7.07). 4-fold increased risk for ductal BC among exposed; only 13 cases	Limited sample size; mostly nulliparous women
Large Danish cohort (Guleria et al.) [36]	2019	1.3 million women	Progesterone	Median 20.9 years	No overall increased BC risk; slight non-significant increase in nulliparous women	Weak trends only
Meta-analysis 2022 (Cullinane et al.) [38]	2022	617,479 women	Progesterone	-	OR 1.11, 95% CI 0.76–1.61; not significant	High heterogeneity (I^2^ = 86%)
ASRM guideline [59]	2024	-	Progesterone in ART	-	No increased BC risk	Evidence-based recommendation. Women should be informed that there does not appear to be an increased risk of breast cancer associated with assisted reproductive technology. Prolonged (>10 cycles) of clomiphene should be avoided (strength of evidence, B; strength of recommendation, weak/moderate).
BRCA1/2 carriers (Kotsopoulos, Librach et al.) [40]	2008	4,994	Progesterone	6.5 years	Unknown; potential concern due to hormone-sensitive tissue. ART is usually associated with increases in circulating endogenous estrogen and progesterone; there is concern that supraphysiologic increases in these hormones might be mitogenic in the breast and that prolonged exposure may increase breast cancer risk by stimulating breast epithelial proliferation.	Individualized approach advised

### Subgroup-specific risk modifiers and methodological interpretation

Across the literature, the most consistent signals of increased breast cancer risk are not observed in the overall ART-exposed population, but rather in selected subgroups. These include nulliparous or nulligravid women, women undergoing repeated or high-dose stimulation cycles, women treated at older reproductive ages, and women with a family history or hereditary predisposition to breast cancer. However, these subgroup findings are not uniform across studies and are often based on small numbers of exposed cases.

Several methodological issues may explain part of the heterogeneity. First, infertility itself may be associated with endocrine and metabolic risk factors that are difficult to disentangle from treatment effects. Second, ART-treated women frequently undergo closer medical surveillance than the general population, which may increase the detection of early or in situ breast cancer. Third, successful ART may be followed by recent childbirth, which is itself associated with a transient increase in breast cancer risk; if ART-treated parous women are compared with a general population that includes both parous and nulliparous women, risk estimates may be biased upward. Finally, many studies lack detailed information on cumulative dose, number of cycles, treatment protocol and time since last birth.

For these reasons, studies using infertile untreated women as the comparator group, adjusting for parity and time since childbirth, and relying on the most recent or most comprehensive follow-up from the same cohort provide the most reliable estimates for clinical interpretation.

## Conclusions

Current evidence does not support a consistent overall increase in breast cancer risk after ART or fertility drug exposure. Large population-based studies and recent meta-analyses are generally reassuring, particularly when ART-treated women are compared with infertile untreated women rather than with the general population.

Some studies have reported modest increases in breast cancer risk in specific subgroups, including nulliparous or nulligravid women, women exposed to repeated or high-dose stimulation cycles, women treated at older reproductive ages, and those with a family history or hereditary predisposition to breast cancer. However, these findings are not consistent across studies and are often limited by small subgroup numbers, heterogeneous exposure definitions, incomplete dose-data information, and residual confounding by infertility-related factors.

The interpretation of short-term increases in breast cancer incidence after successful ART also requires caution, because recent childbirth itself may transiently increase breast cancer risk. Comparisons with the general female population may therefore overestimate ART-related risk if parity, time since last birth, and intensity of medical surveillance are not adequately considered.

Overall, the available evidence is reassuring, but it does not completely exclude a small risk increase in selected high-risk subgroups. Future studies should prioritize long-term follow-up, accurate drug-specific and dose-specific exposure data, appropriate infertile control groups, and adjustment for parity, time since childbirth, family history, and genetic predisposition. Until more definitive evidence is available, individualized counseling and risk-adapted breast surveillance may be considered for women with additional breast cancer risk factors undergoing ART.

## Data Availability

No datasets were generated or analysed during the current study.

## Abbreviations

ART: Assisted reproductive technologies
BC: Breast cancer
CC: Clomiphene citrate
hMG: Human Menopausal Gonadotropin
FSH: Follicle-Stimulating Hormone
LH: Luteinizing Hormone
